# The assessment of caregiver burden and associated factors on the primary family caregivers for patients with moyamoya disease: a cross-sectional study

**DOI:** 10.3389/fpsyt.2025.1688617

**Published:** 2025-10-15

**Authors:** Meng Li, Jianhua Li, Hanjiao Liu, Zhongjie Pan, Bohao Zhang, Wenling Zhang, Jiaxin Li, Kezhen Yang, Yuandong Yu, Tie Yang, Dongsen Lv, Ning Zhang, Shen Li, Qiong Li, Ruipeng Song, Junxiao Liu, Huawei Li, Junfan Wei

**Affiliations:** ^1^ Nursing Department, The Third People’s Hospital of Henan Province, Zhengzhou, China; ^2^ Nursing Department, Nanjing Central Hospital, Nanjing, China; ^3^ Nursing Department, Shenzhen Hospital of Integrated Traditional Chinese and Western Medicine, Shenzhen, China; ^4^ School of Medicine, Zhengzhou University of Industrial Technology, Zhengzhou, China; ^5^ Department of Oncology, The First Affiliated Hospital of Zhengzhou University, Zhengzhou, China; ^6^ Surgical Anesthesia Department, Henan Provincial People’s Hospital, Zhengzhou, China; ^7^ School of Medicine, Xi’an International University, Xi’an, China; ^8^ School of Nursing, Xinxiang Medical University, Xinxiang, China; ^9^ School of Rehabilitation, Henan Vocational College of Tuina, Luoyang, China; ^10^ Department of Operating Room, The Affiliated Cancer Hospital of Zhengzhou University, Zhengzhou, China; ^11^ The Seventh Clinical Medical College, Guangzhou University of Chinese Medicine, Shenzhen, China

**Keywords:** caregiver burden, moyamoya disease, activities of daily living, illness uncertainty, family caregiver

## Abstract

**Background:**

Caring for moyamoya disease (MMD) patients is a demanding, prolonged, and continuous responsibility, often resulting in a significant caregiver burden for primary family caregivers. Considering the enormous challenges faced by the primary caregiver in a family and the serious consequences of the caregiver burden, it becomes crucial to know current status and assess the factors associated with the caregiver burden.

**Objective:**

This study aimed to assess caregiver burden among primary family caregivers of MMD patients and examine its association with patients’ Activities of Daily Living (ADL), caregivers’ socio-demographic characteristics, and illness uncertainty.

**Method:**

A cross-sectional survey using convenience sampling was conducted in China. Data collection conducted from January to July 2024 at two tertiary hospitals in Henan province, China. A socio-demographic characteristics questionnaire, the Chinese version of Barthel Index scale, the Chinese version of Zarit burden interview scale, and the Chinese versions of Mishel illness uncertainty scale for family member were used to perform this research. The collected data were analyzed using SPSS 24.0 statistical software.

**Result:**

A total of 287 primary caregiver of patients with MMD were recruited in this survey. Of the 287 primary family caregivers of patients with MMD, 44 (15.33%) experienced mild burden, 106 (36.93%) experienced moderate burden, and 137 (47.74%) experienced severe burden. Multiple linear regression analysis revealed that marriage status (*β* = 0.079, *P* = 0.027), average monthly income (*β* = -0.515, *P*<0.001), daily care hours(*β* = 0.138, *P*<0.001), BI (*β* = 0.243, *P*<0.001) and illness uncertainty (*β* = 0.255, *P*<0.001) are associated factors with caregiver burden.

**Conclusion:**

This study found that nearly half of the primary family caregivers of patients with MMD had severe levels of caregiver burden, which is influenced by several factors. These findings contribute to a better understanding of the challenges faced by caregivers of MMD patients and may inform future research, clinical assessments, and supportive resource planning.

## 1 Introduction

Moyamoya disease (MMD) is a rare, chronic, and progressive cerebrovascular disorder (1) first proposed by Japanese surgeons in 1957 (2). MMD exhibits regional differences in its global incidence. It is more prevalent in East Asia, particularly in Japan and South Korea, where the annual incidence rates range from 0.5 to 1.5 per 100,000 people (3), compared to as low as 0.1 per 100,000 in North America (3). Specifically, Japan reports an incidence of 0.94/100000, while South Korea’s is 2.3/100,000 (4). In Taiwan, the average incidence is 0.15/100000 (5), and a study in Nanjing reported an incidence of 3.92/100000 between 2000 and 2007 (6), which is lower than in Japan and South Korea. However, comprehensive national epidemiological studies on MMD in mainland China remain scarce. It is characterized by progressive stenosis or occlusion of the intracranial internal carotid arteries and their proximal branches, potentially leading to ischemic or hemorrhagic strokes and seizures (7). Furthermore, even asymptomatic patients with MMD may experience cognitive impairments, particularly in areas such as intelligence, spatial ability, verbal working memory, and numerical processing (8–10). Due to its disabling nature, MMD often results in reduced activities of daily living (ADLs), limited social participation (11), and serious impacts on physical health, mental well-being, and family functioning (12).

In many regions, especially where access to comprehensive public health services is limited, family members—most commonly spouses or adult children—serve as the primary caregivers for patients with MMD (13). These caregivers are deeply involved in the entire treatment process, not only providing daily care and emotional support but also playing a crucial role in making treatment decisions (14). However, caring for MMD patients is a long-term and demanding responsibility, placing a considerable burden on caregivers both physically and psychologically (15). The concept of “caregiver burden” refers to the subjective and multidimensional strain experienced during caregiving, encompassing emotional distress, physical fatigue, economic pressure, and social constraints (16–18).

In recent years, the problem of caregiver burden, especially that of the family’s primary caregiver, has received increasing attention from scholars in the health care field (9, 20). Research has shown that caregiver burden issues can lead to a number of negative consequences. Mental health problems are one of the most prevalent consequences, with caregivers experiencing significantly higher rates of anxiety, depression, and emotional exhaustion (19). These psychological burdens not only reduce caregivers’ quality of life, but may also negatively impact their ability to care. Deteriorating physical health is also a common problem, and chronic physical exertion and caregiving-related stress may increase caregivers’ risk of developing chronic diseases (e.g., hypertension, cardiovascular disease) (20). Additionally, financial burden is another key consequence, especially when caregiving requires a significant time commitment (21). At the same time, social isolation is a common challenge for primary caregivers of people with chronic illnesses, and long-term caregiver burden may reduce their opportunities for socialization, leading to feelings of loneliness and lack of social support (22). More importantly, research has also found that caregiver burden can have a knock-on effect, such as decreasing the quality of care for patients, leading to tensions in the caregiver-patient relationship (23).

Considering the enormous challenges faced by the primary caregiver in a family and the serious consequences of the caregiver burden, it becomes crucial to assess the factors associated with the caregiver burden. Previous studies for other diseases have emphasized that the socio-demographic characteristics of the patient’s primary caregiver, such as age, place of residence, income level, etc., are key factors influencing the caregiver burden (24, 25). Another factor is the Activities of Daily Living (ADL), referring to basic tasks performed daily to sustain life and adapt to the environment, serving as a key measure of a patient’s independence (26). ADL has been widely supported by research to be significantly associated with caregiver burden (27, 28). Specifically, the higher the patient’s ability to activity of daily living, the lower the burden of care on the patient’s primary family caregiver. In addition, illness uncertainty is an important cognitive and emotional factor in caregiver psychological burden. According to Mishel’s theory of uncertainty in illness, uncertainty stems from ambiguous perceptions of the disease condition, course of treatment, and outcome, a state that triggers anxiety and emotional exhaustion in caregivers (29). Research has found that illness uncertainty not only significantly increases caregiver psychological distress, but may also further increase caregiver perceived burden by influencing decision-making processes and patient interactions (30). Particularly in chronic care, caregiver uncertainty about disease progression can lead to high levels of emotional instability and decision-making dilemmas, which can significantly affect the quality of care and caregiver physical and mental health (31). While these factors have been studied in various populations, little is known about their role in caregiving for MMD specifically.

Although the caregiver burden has been extensively studied in research on disorders such as stroke and cancer, the caregiving experience for MMD patients presents unique challenges, making dedicated research on this population essential (32). Unlike stroke, which typically has an acute and sudden onset with a relatively predictable recovery trajectory, MMD is a chronic, progressive, and unpredictable cerebrovascular disease (1). Recurrent ischemic or hemorrhagic events, coupled with cognitive impairment and functional decline, create long-term uncertainty for caregivers (33). Unlike cancer care, where treatment pathways and prognosis information are typically well-defined, MMD lacks clear treatment outcomes and exhibits significant variability in clinical progression (34). This uncertainty not only intensifies emotional strain but also complicates decision-making for caregivers, as they must continuously adapt to fluctuating patient needs without clear expectations of disease course (32). Furthermore, MMD often affects younger patients compared to stroke or cancer, thereby placing caregiving responsibilities on families during critical economic and social stages of life (35). These unique features—chronic unpredictability, cognitive involvement, and younger age of onset—highlight why caregivers of MMD patients may experience a higher and qualitatively different burden than caregivers in other conditions, underscoring the importance of this study (32).

Therefore, the purpose of this study was to explore the current caregiver burden of primary family caregivers of patients with MMD and to examine the impact of MMD patients’ ADLs, their primary caregiver’s socio-demographic factors, and illness uncertainty on caregiver burden, in order to better understand the primary caregiver burden of MMD patients. By addressing this gap in the literature, our study provides valuable insights into the unique challenges faced by primary caregivers of people with MMD. Additionally, these findings may provide information for evidence-based strategies to reduce caregiver burden and ultimately improve the quality of in-home care for patients as well as the quality of life for both patients and caregivers.

## 2 Methods

### 2.1 Study design

Given the rarity of MMD and the limited number of eligible caregivers, convenience sampling was employed to gain preliminary insights into relevant aspects of the caregiving process (36). This approach aligns with the exploratory nature of the study and is consistent with the objective of understanding the unique challenges faced by caregivers of individuals with MMD (37). A cross-sectional survey using convenience sampling (38) was conducted between January and July 2024 in two tertiary hospitals located in Henan Province, China. The present study adheres to the Strengthening the Reporting of Observational Studies in Epidemiology (STROBE) reporting guideline.

### 2.2 Participants

Calculating the sample size for this study following the principles of sample size calculation in the Kendall’s cross-sectional survey (39), n = independent variable × (5~10) (40). For this study, we used a total of four scales consisting of 26 independent variables, 10 of which were from the socio-demographic characteristics questionnaire (including age, gender, marital status, level of education, employment status, average monthly income, habitation, presence of chronic disease, daily care hours and assistance from others), and the other 10 independent variables were from the Chinese version of Barthel Index, 2 from the Chinese version of Zarit Caregiver Burden Interview Scale and 4 from the Chinese versions of Mishel Uncertainty in Illness Scale for Family Member. Considering the 20% invalid questionnaires, the minimum sample size required for this study was 163 to 325.

Participants were recruited using convenience sampling from two tertiary hospitals in Zhengzhou, Henan Province, China. The inclusion criteria for primary family caregivers were as follows: (1) assume the role of primary family caregivers for patients diagnosed with MMD or unilateral MMD by a clinician according to the criteria set by the Neuroradiology Committee (41). (2) Older than 18 years old. (3) The primary family caregivers of the patients were responsible for the main care tasks and took the longest care time. (4) Participating in this study voluntarily. On the other hand, the exclusion criteria for primary family caregivers were: (1) primary family caregivers have serious cognitive dysfunction. (2) Caregivers are nannies, caregivers, or salaried workers. (3) The primary family caregivers has language communication barriers and unable to complete the questionnaire independently or under the guidance.

### 2.3 Instruments

#### 2.3.1 Socio-demographic characteristics questionnaire

A self-designed questionnaire was used to collect socio-demographic characteristics of the respondents, consisting of 10 questions on age, gender, marital status, level of education, employment status, average monthly income, habitation, presence of chronic disease, daily care hours and assistance from others.

#### 2.3.2 The Chinese version of Barthel index scale

This scale was developed by Florence Mahoney and Dorothy Barthel (42). It is mainly used to evaluate the activity of patients’ ADLs. It contains 10 items, 8 items are self-care activities (eating, grooming, going to the toilet, bathing, dressing, transferring, and defecation control), and 2 items are action related activities (walking on the ground or in a wheelchair for 50m, going up and down stairs. Among the 10 items, each item has different degrees of scores, including 6 items with 3 degree scores (0∼10 points), 2 items with 2 degree scores (0∼5 points), and 2 items with 4 degree scores (0∼15 points). The total score of the scale is 100 points. The higher the score, the better the independence and the less dependence. The evaluation method involves direct observation or interviewing patients and primary family caregivers for scoring. According to the total score, activity of daily living is classified as intact, no dependency required (100 score), mild dependence, some need to be taken care of by others (61∼99 score), moderate dependence, most need to be taken care of by others (41∼60 score). Less than 40 points means heavy dependence, all need help. Research has shown that the Chinese version of the BI scale demonstrates strong validity and reliability (43). It has also shown excellent reliability, with high test-retest and inter-rater reliability (kappa values ranging from 0.63 to 1.00), as well as internal consistency (Cronbach’s α = 0.93). Data were collected via face-to-face interviews conducted by the researchers. In the present study, the Cronbach’s α of this scale was 0.854.

#### 2.3.3 The Chinese version of Zarit burden interview scale

This scale was compiled by Zarit et al. (18) to investigate the care burden of caregivers. The Chinese version of 22-item Zarit Burden Interview Scale was translated by Lie Wang et al. (44) and shown accepted reliability and validity. The scale has 22 items and is a two-dimension (personal burden and responsibility burden) scale. The scale is self-rated on a 5-point Likert scale with 0 = almost never and 4 = always. The final score of this scale is the sum of the item scores, with a higher total score indicating a higher level of care burden among the primary family caregivers. In the present study, the Cronbach’s α of this scale was 0.97. A total scale score of less than 21 was categorized as no or mild burden, 21 to 39 chants were categorized as moderate burden, and 40 and above were categorized as severe burden.

#### 2.3.4 The Chinese version of Mishel illness uncertainty scale for family member

This scale was developed by Mishel, an American nursing expert, based on the theory of illness uncertainty (45) to measure the illness uncertainty of the patient’s primary family caregivers. The Chinese version of illness uncertainty scale for primary family caregivers translated by Wenying Wang (46) et al. It is often used to measure illness uncertainty of family members in patients’ with chronic diseases (e.g., breast cancer, stroke, etc.) in previous studies (47). This scale consists of 30 items, which fit into four different dimensions: uncertainty, lack of information, complexity and unpredictability. All the items in this study were scored by the Likert 5 method (1 = strongly disagree, 5 = strongly agree). 20 positive scores and 10 negative scores. The total scores ranged from 30 to 150. In one previous study, the degree of illness uncertainty of primary family caregivers was graded according to the total score, respectively 30~70 being low, 71~111 being moderate and 112~150 being high. The higher the score, the higher the level of illness uncertainty. The Cronbach’s α of the original MUIS-FM is 0.91 (47). In the present study, the Cronbach’s α was 0.92.

### 2.4 Data collection

The present study was a cross-sectional survey with convenience sampling, and data were collected from February to August 2024 in two tertiary hospitals in Henan Province. The researcher distributed the paper version of the questionnaire face-to-face to the participants, who answered it alone in a quiet environment. This questionnaire consisted of four scales, including socio-demographic characteristics questionnaire, BI, ZCBI, and MUIS-FM. The questionnaires were filled out anonymously, and participants were not allowed to leave until their answers were checked by the researcher for any omissions; if there were omissions, the researcher would remind the participants to complete the questionnaire. Two researchers independently collected paper questionnaires and input them into a computer; the causes of the differences were identified and resolved in a timely manner. Participants were recruited from outpatient clinics and inpatient wards at both hospitals. Eligible participants were briefed about the study objectives and provided with information regarding their rights as participants. Participants were assured of the confidentiality and anonymity of their responses, and data were securely stored and accessible only to the research team. Ethical approval for the study was obtained from the institutional review board before investigation, and written informed consent was obtained from each participant.

### 2.5 Data analysis

Data were analyzed by using SPSS 26.0 (IBM Company, Armonk, NY, USA). The level of statistical significance was set at *P* < 0.05. In the descriptive statistics section, categorical variables are presented in numbers and percentages. Kolmogorov-Smirnov test was used for normality test. If the continuous variable conforms to the normal distribution, it is displayed by the mean ± standard deviation, and if it does not conform to the normal distribution, it is displayed by the median and interquartile range (IQR). Spearman correlation analysis was calculated to examine associations between BI level, ZBI score, and illness uncertainty level. Two-sided t-test and one-way analysis of variance (ANOVA) and linear regression analysis (enter method) were conducted to analysis explore independent factors related to caregiver burden. Variables that were significant in the univariate analysis were used as independent variables, and caregiver burden was entered into the multiple linear regression model as the dependent variable. With significant correlations, Barthel Index scores and illness uncertainty scores were included in the regression model after transforming them into categorical variables according to their hierarchical approach, while caregiver burden scores were included in the regression model according to their own scores as continuous variables.

### 2.6 Ethical considerations

Ethical approval for this study was obtained from the third People’s Hospital of Henan Province in Zhengzhou, China (Ethical Review NO.: 2024-SZSYKY-002). Prior to data collection, written informed consent was obtained from all participants and they were provided with a written explanation of the purpose and procedures of the study. The researcher informed participants that they could withdraw from the study at any time. Confidentiality and anonymity were followed throughout the study. All original versions of the paper questionnaires were kept in the exclusive safekeeping of a single researcher, and any researcher within the research team who wanted to access the questionnaires was required to specify their use to obtain the consent of the corresponding author.

## 3 Results

### 3.1 Social-demographic characteristics of participants and comparison of different variables on caregiver burden

A total of 420 primary family caregivers were invited to participate in this study, and only 304 of them agreed to participate in this study, but 14 of them had incomplete questionnaires, and 3 of them did not fill in the questionnaires. Finally, only 287 valid questionnaires were collected, and the effective recovery rate was 68.3%. 149 (51.92%) of the 287 participants were younger than 30 years old. There were 151 males, accounting for 52.61% of the total participants. In terms of marital status, most of the participants were unmarried, with 271 individuals, accounting for 94.43% of the total. Among the participants in this study, 60 (20.91%) holders of college diplomas. 125 (43.55%) participants did not work. The average monthly income of most participants were 1001~3000 yuan (34.89%). 167 (58.19%) of the participants lived in the city. 88 (30.66%) of the 287 did not have a chronic disease, while the rest had a chronic disease. Most participants (117; 40.77%) took care of patients less than 4 hours a day, and most of the participants (168; 58.54%) were assisted by other caregivers.

In our study, assessing the caregiver burden of primary family caregivers providing care to MMD patients, the average total ZBI score was found to be (38.24 ± 16.26). Of the 287 primary family caregivers of patients with MMD, 44 (15.33%) experienced mild burden, 106 (36.93%) experienced moderate burden, and 137 (47.74%) experienced severe burden. Significant differences were found in caregiver burden by socio-demographic characteristics such as marital status, level of education, average monthly income, daily care hours and assistance from other caregivers (respectively; *P* = 0.002, *P =* 0.007, *P* = <0.001, *P* <0.001, *P* <0.001). On the contrary, there were no significant differences in caregiver burden score according to the primary family caregiver’s age (*P* = 0.205), gender (*P* = 0.113), employment status (*P* = 0.513), habitation (*P* = 0.107) and presence of chronic disease (*P* = 0.280). See **Table 1** for details.

**Table 1:** Social-demographic characteristics of participants and comparison of different variables on caregiver burden (N = 287).

Items		N	%	M ± SD	T/f	*P*
Age	18-29	149	51.92	40.12 ± 17.29	1.490	0.205
	30-39	126	43.90	36.15 ± 15.20		
	40-49	9	3.14	34.44 ± 12.55		
	50-59	1	0.35	24 ± 0.0		
	≥60	2	0.70	44.5 ± 7.78		
Gender	Male	151	52.61	36.73 ± 16.31	-1.588	0.113
	Female	136	47.39	39.78 ± 16.19		
Marital status	Unmarried	16	5.57	26.19 ± 13.03	9.592**	0.002
	Married	271	94.43	38.96 ± 16.17		
Level of education	Primary school education	41	14.29	40.90 ± 16.32	3.232**	0.007
	Junior high school education	50	17.42	43.84 ± 15.00		
	High school education	54	18.82	33.69 ± 15.73		
	Junior college diploma	60	20.91	34.28 ± 15.25		
	Bachelor’s degree	49	17.07	38.57 ± 16.72		
	Master’s degree	33	11.50	40.64 ± 17.33		
Employment status	Working	162	56.45	38.80 ± 16.75	0.654	0.513
	Not working	125	43.55	37.53 ± 15.65		
Average monthly income (in CNY)	≤ 1000 yuan	34	11.85	60.44 ± 9.95	104.839***	<0.001
	1001 ~ 3000 yuan	100	34.84	46.34 ± 9.46		
	3001 ~ 5000 yuan	57	19.86	30.40 ± 10.73		
	>5000 yuan	96	33.45	26.60 ± 13.48		
Habitation	Countryside	69	24.04	34.64 ± 14.61	2.253	0.107
	Township	51	17.77	39.43 ± 17.70		
	City	167	58.19	39.37 ± 16.33		
Presence of chronic disease	None	88	30.66	36.25 ± 17.33	1.283	0.280
	One	76	26.48	41.11 ± 15.20		
	Two	67	23.34	37.46 ± 15.25		
	Three or more	56	19.51	38.43 ± 16.96		
Daily care hours	<4 h/Day	117	40.77	30.98 ± 16.01	27.664***	<0.001
	4–8 h/Day	91	31.71	40.11 ± 14.66		
	>8h/Day	79	27.53	46.85 ± 13.51		
Assistance from other caregivers	Yes	168	58.54	41.86 ± 15.12	-21.504***	<0.001
	No	119	41.46	33.13 ± 16.50		

**P < 0.01; M, mean. SD, standard deviation.

### 3.2 Spearman correlation analysis of caregiver burden score, BI level and illness uncertainty level in MMD patients’ primary family caregivers

According to spearman correlation analysis, caregiver burden was significantly correlated with Barthel Index (r = 0.525**, *P* <0.001) and was significantly correlated with illness uncertainty (r = 0.521**, *P* <0.001). Further details can be found in **Table 2**.

**Table 2:** Spearman correlation analysis of caregiver burden score, BI level and illness uncertainty level in MMD patients’ primary family caregivers.

Variables	Caregiver burden	Barthel index (BI)	Illness uncertainty
Caregiver Burden	1		
Barthel Index (BI)	0.525**	1	
Illness uncertainty	0.521**	0.284**	1

**P < 0.01.

### 3.3 Multivariate linear regression analysis of factors influencing the caregiver burden MMD patients’ primary family caregiver

Multicollinearity among the independent variables was identified to review the basic assumptions of the regression model. Considering that the variation inflation factor (VIF) was 10 or less and tolerance was 0.1 or above, there was no evident issue in terms of multicollinearity. As the Durbin-Watson statistic was 1.818, which is close to 2.0, there was no autocorrelation, indicating that the error terms were independent of each other.

The items with statistical significance (*P*<0.05) in the univariate analysis of primary family caregiver burden based on social-demographic characteristics and correlation analysis used as independent variables, and the caregiver burden was used as the dependent variable for enter multiple linear regression analysis.

As shown in **Table 3**, the results showed that the model explained 67.4% of the total variance in the variance of caregivers burden, which was statistically significant (F = 85.611, *P*<0.001). The results showed that marriage status (*β* = 0.079, *P* = 0.027), average monthly income (*β* = -0.515, *P*<0.001), daily care hours(*β* = 0.138, *P*<0.001), BI (*β* = 0.243, *P*<0.001) and illness uncertainty (*β* = 0.255, *P*<0.001) are contributing factors to the caregiver burden among the primary family caregivers of MMD patients.

**Table 3:** Hierarchical regression analysis for factors associated with the caregiver burden for primary family caregivers.

Factors	B	SE	*β*	T	*P* value	95%CI		Tolerance	VIF
						Lower	Upper		
Constant	20.072	5.750		3.490	<0.001	8.752	31.391		
Marital status	5.622	2.530	0.079	2.222	0.027	0.641	10.603	0.890	1.123
Level of education	-0.210	0.351	-0.020	-0.598	0.551	-0.901	0.481	0.981	1.019
Average monthly income(in CNY)	-7.999	0.599	-0.515	-13.353	<0.001	-9.178	-6.820	0.765	1.308
Daily care hours	2.742	0.721	0.138	3.804	<0.001	1.323	4.161	0.868	1.152
Assistance from other caregivers	-0.354	1.171	-0.011	-0.303	0.762	-2.660	1.951	0.901	1.110
Barthel index	4.494	0.687	0.243	6.544	<0.001	3.142	5.846	0.825	1.212
Illness uncertainty	8.057	1.385	0.225	5.817	<0.001	5.331	10.783	0.763	1.311

R = 0.826, R^2^ = 0.682, adjusted R^2^ = 0.674; B = partial regression coefficient, SE = standard error, *β =* standardized regression coefficients, 95%CI = 95% Confidence interval for B.

## 4 Discussion

As a progressive cerebrovascular disease, the progression of MMD can lead to extremely serious consequences, which brings psychological, physiological and economic challenges to not only the patients themselves but also their main family caregivers (3, 4). This poses a potential threat to the well-being of family members who provide care for people with MMD. This study aimed to explore the related factors contributing to caregiver burden in primary family caregivers of patients. Results indicate that nearly half of primary caregivers for MMD patients experience significant caregiver burden. Marital status, average monthly income, daily care hours, BI, and illness uncertainty are associated factors influencing caregiver burden. These results offer valuable insights into the unique challenges faced by caregivers of MMD patients.

Our research found that the gender of the caregivers was majority male (52.61%). This finding was similar to studies conducted by Parvizi, M (25). et al. However, other studies have reported that most caregivers are female (48, 49). This may be related to a variety of factors such as the source of the sample, cultural background and social structure. Future research could further explore the causes of gender differences among caregivers and examine whether this trend shifts as social structures change.

At present, there seems to be little relevant research on caregiver burden for primary family caregivers of MMD patients. Most previous studies tend to focus on cerebrovascular diseases primary family caregivers in general, such as stroke (48, 50) and cerebral hemorrhage (51). Considering that MMD is also a kind of cerebrovascular disease, the results of these studies on the primary family caregivers of cerebrovascular disease may have certain reference significance for the current research.

One of the key findings of this study was the evidence that the primary family caregivers of patients with MMD experience significant caregiver burdens. The results showed that the mean caregiver burden score of primary family caregivers of patients with MMD was (38.24 ± 16.26), which was slightly higher than (32.80 ± 11.97) obtained by Kazemi A (48) et al. who conducted a survey of caregivers of elderly stroke patients, suggest that caregivers of MMD patients may face an even greater burden. What’s more, the results revealed that 47.74% of the caregivers in this study reported severe burden, almost half of them, and the remaining caregivers experiencing moderate to mild burden. However, the findings of Kazemi A (48) et al. showed that only 0.9% caregiver was under severe burden. In another study, Kumar R (52) et al. reported that 37% of caregivers of stroke patients experienced a severe caregiver burden, also lower than in this study. The difference in caregiver burden between stroke and primary caregivers of MMD suggests that caregivers of patients with moyamoya disease may face a greater burden. While both MMD and stroke are cerebrovascular diseases, MMD typically presents with more gradual and unpredictable symptom progression, which can exacerbate caregiver stress. The continuous uncertainty regarding disease progression may contribute to a higher caregiver burden in MMD compared to stroke, where some patients may have more stable recovery trajectories. Given these unique challenges, healthcare providers can play a significant role in reducing caregiver burden. Actionable recommendations include offering continuous education on the disease progression and potential complications, which can help caregivers manage expectations and make informed decisions. Additionally, healthcare teams should provide psychological support for caregivers, acknowledging the emotional strain and offering resources to help them cope with stress. Creating individualized care plans that address the fluctuating needs of MMD patients and regularly updating caregivers about the patient’s condition can also help reduce feelings of uncertainty. Finally, encouraging support groups and peer networks can allow caregivers to share experiences and receive emotional support from others in similar situations.

Another key findings of this study was the significant correlation between caregiver burden and various socio-demographic factors, including marital status, level of education, average monthly income, daily care hours, and the presence of assistance from other caregivers. These results align with previous studies that have indicated the significant role socio-demographic factors play in impacting caregiver experiences.

Consistent with previous studies, our results indicate that marital status plays an important role in caregiver burden. Married caregivers often report higher levels of burden, likely due to the combined emotional and logistical demands of caregiving (53). In contrast, unmarried caregivers may face additional difficulties, such as the lack of emotional or financial support, which exacerbates their caregiver burden.

Further, socio-economic factors such as income level and education were identified as important predictors of caregiver burden. Caregivers with higher education levels and better financial stability generally report lower levels of caregiver stress (54). These caregivers are better equipped to manage the financial and emotional challenges of caregiving, often benefiting from better coping strategies and access to healthcare resources (55).

In addition, income and education emerged as significant predictors of caregiver burden. Caregivers with higher education levels and better financial stability tend to experience less burden, likely due to improved coping mechanisms and greater access to resources (55). These findings corroborate prior studies that indicate that economic challenges contribute to increased caregiver burden (24, 56).

Caregiving hours also played a significant role in caregiver burden. This may be caused by the fact that caregivers who spend longer hours providing care experience higher levels of physical and emotional exhaustion, and burnout leads to additional burdens. This is consistent with the broader caregiving literature, which has shown that continuous caregiving without adequate respite leads to higher stress and poorer health outcomes (57, 58). The findings further emphasize the importance of respite care and social support in alleviating the burden on caregivers.

Consistent with the results of other previous studies, the present study found that caregivers of patients with lower BI scores report significantly higher levels of caregiver burden (25). The increased burden can be attributed to the fact that caregivers often have to assist with basic tasks such as bathing, dressing, and feeding, which may demand more time, energy, and emotional involvement (48). Further, caregivers of patients with severe impairments often experience “caregiver burnout,” characterized by physical exhaustion, emotional distress, and a sense of helplessness.

We also found illness uncertainty to be a major contributor to caregiver burden. This is especially relevant for caregivers of MMD patients, a disease characterized by unpredictable progression and varying clinical outcomes. As noted in previous studies, illness uncertainty contributes to caregiver anxiety and emotional strain, as caregivers often struggle with making long-term care plans (55). Similar findings have been reported for caregivers of patients with chronic diseases such as cancer, where uncertainty about treatment outcomes and patient recovery can exacerbate emotional distress (59). The strong correlation between illness uncertainty and caregiver burden in this study underscores the need for clear communication from healthcare providers regarding disease progression and expected outcomes. Providing caregivers with more information and guidance could help reduce their anxiety and better prepare them for the future.

### 4.1 Limitations

Although we have tried our best to refine the research design of this study, there are still several limitations that are difficult to avoid. First of all, a potential limitation is the sample’s demographic diversity, as participants were primarily selected from specific geographic areas using convenience sampling, which may introduce selection bias and limit the generalizability of the results. Additionally, the cross-sectional nature of the survey may restrict the ability to infer causality from the associations observed between ADL, caregiver burden, and illness uncertainty. Furthermore, the study focused on primary family caregivers, which may overlook the broader family dynamics involved in caregiving, such as the role of siblings or extended family members.

## 5 Conclusion

This study found that nearly half of the primary family caregivers of patients with MMD had severe levels of caregiver burden, which is influenced by several factors including the patient’s ADL, the caregiver’s socio-demographic characteristics (marital status, average monthly income, daily care hours) and illness uncertainty. To reduce this burden, we recommend integrating regular caregiver assessments into routine care to better identify and address their needs. Additionally, these findings underscore the need for targeted interventions that address the specific challenges faced by primary family caregivers of MMD patients, particularly in terms of managing uncertainty and providing emotional and practical support. Health professionals should adopt a more holistic approach to caregiving, incorporating strategies to reduce the burden to sustain quality care for MMD patients.

For future research, we recommend employing longitudinal study designs to better understand how caregiver burden evolves over time and how different support systems influence the caregiving process and explore the effectiveness of targeted interventions to support caregivers. Furthermore, broadening the scope to encompass a wider family perspective—such as the roles of extended family members—could provide additional insights into the dynamics of MMD family caregiving.

## 6 Relevance to clinical practice

The findings of this study hold significant implications for clinical practice, particularly in the context of supporting family caregivers of patients with chronic conditions like MMD. It is crucial for healthcare professionals to be aware of the substantial care burden experienced by primary caregivers and the associated psychological stress, such as illness uncertainty. Incorporating routine screenings for caregiver burden and illness uncertainty into clinical settings could help identify caregivers in need of support. Healthcare providers should work closely with caregivers, offering guidance and resources to manage caregiving tasks more effectively. Additionally, policies that include psychological support and social services for caregivers are essential to enhance the overall care system and ensure that caregivers’ needs are addressed in tandem with those of the patients.

## Data Availability

The raw data supporting the conclusions of this article will be made available by the authors, without undue reservation.
